# Broth Microdilution and Gradient Diffusion Strips vs. Reference Agar Dilution Method: First Evaluation for *Clostridiales* Species Antimicrobial Susceptibility Testing

**DOI:** 10.3390/antibiotics10080975

**Published:** 2021-08-12

**Authors:** Florian Baquer, Asma Ali Sawan, Michel Auzou, Antoine Grillon, Benoît Jaulhac, Olivier Join-Lambert, Pierre H. Boyer

**Affiliations:** 1 Laboratory of Bacteriology, Strasbourg University Hospital, F-67000 Strasbourg, France; baquer.florian@gmail.com (F.B.); asma.a.sawan@gmail.com (A.A.S.); a.grillon@unistra.fr (A.G.); jaulhac@unistra.fr (B.J.); 2 Department of Medical Microbiology and Parasitology, Faculty of Medicine, King Abdulaziz University, Jeddah 21589, Saudi Arabia; 3 Research Group on Microbial Adaptation GRAM 2.0, Department of Microbiology and Hygiene, Caen University Hospital of Caen, UniCaen-UniRouen, F-14033 Caen, France; auzou-m@chu-caen.fr (M.A.); joinlambert-o@chu-caen.fr (O.J.-L.); 4 Institute of Bacteriology, University of Strasbourg, UR7290, ITI InnoVec, Fédération de Médecine Translationnelle de Strasbourg, 3 rue Koeberlé, F-67000 Strasbourg, France

**Keywords:** anaerobe, antimicrobial susceptibility testing, broth microdilution, gradient diffusion method, *Clostridiales*

## Abstract

Antimicrobial susceptibility testing of anaerobes is challenging. Because MIC determination is recommended by both CLSI and EUCAST, commercial broth microdilution and diffusion strip tests have been developed. The reliability of broth microdilution methods has not been assessed yet using the agar dilution reference method. In this work, we evaluated two broth microdilution kits (MICRONAUT-S Anaerobes^®^ MIC and Sensititre Anaerobe MIC^®^) and one gradient diffusion strip method (Liofilchem^®^) for antimicrobial susceptibility testing of 47 *Clostridiales* isolates (*Clostridium, Clostridioides* and *Hungatella* species) using the agar dilution method as a reference. The evaluation focused on comparing six antimicrobial molecules available in both microdilution kits. Analytical performances were evaluated according to the Food and Drug Administration (FDA) recommendations. Essential agreements (EA) and categorical agreements (CA) varied greatly according to the molecule and the evaluated method. Vancomycin had values of essential and categorical agreements above 90% for the three methods. The CA fulfilled the FDA criteria for three major molecules in the treatment of Gram-positive anaerobic infections (metronidazole, piperacillin/tazobactam and vancomycin). The highest rate of error was observed for clindamycin. Multicenter studies are needed to further validate these results.

## 1. Introduction

Anaerobes are major components of the normal bacterial flora mainly found in the digestive tract [1]. They are also part of the genital, oropharyngeal and skin microbiota [1]. Consequently, anaerobes are frequently isolated in the context of endogenous infections [2]. Anaerobes are also responsible for exogenous infections such as gas gangrene [3]. Bacteremia can further complicate these two types of initial infections (endogenous or exogenous), resulting in a high mortality rate, especially if the antibiotic regimen is not effective against anaerobic bacteria [4]. Therefore, their isolation, identification and especially antimicrobial susceptibility testing (AST) are of utmost importance in order to detect antibiotic resistance, which is increasing among these bacteria [5].

To assess the susceptibility of anaerobic bacteria to antimicrobials, different methods are available and were reviewed by Brook et al. [2]. For anaerobes, the agar dilution method (ADM) is recognized as the reference method to determine Minimum Inhibitory Concentration (MIC) values, but this technique is reserved to specialized centers for population studies and is not suitable to routinely use [2]. Broth microdilution (BMD) is also deemed as a reference method, but only for bacteria of the *Bacteroides fragilis* group [1]. For routine AST, the disc diffusion method can be performed according to the Clinical and Laboratory Standards Institute (CLSI) [6] and other scientific societies guidelines (*e.g.,* French Society for Microbiology [7]). However, the disc diffusion method does not provide precise MIC values for a given molecule, which is mandatory to monitor severe infections. Indeed, for *Enterobacterales*, while being determined as ‘susceptible’, isolates with higher MIC values are associated with a higher risk of clinical failure and increased mortality than those with lower MIC values [8]. This could be extended for anaerobes, especially in cases of severe infections. BMD and the gradient diffusion method (GDM) (also called gradient strip method) are two attractive alternatives to ADM in order to determine MICs, these latter alternatives make it easier to determine MICs for a given isolate from a patient.

The GDM usually correlates well with the ADM, even if discrepancies have been evidenced [2,9,10,11]. There are two commercially available BMD kits, the MICRONAUT-S Anaerobes^®^ MIC test (Merlin Diagnostika GmbH, Bornheim, Germany) which is validated for several anaerobes species by the manufacturer, and the Thermo Scientific Sensititre Anaerobe MIC^®^ plate (Trek Diagnostic Systems, Thermo Fisher Scientific, Cleveland, OH, USA) which is validated for the *Bacteroides* spp. bacteria. However, to our knowledge, there are no recent studies comparing these BMD kits to the reference ADM. The Sensititre Anaerobe MIC^®^ plate has been evaluated against the GDM with several Gram-negative isolates [12] and compared to the ATB ANA^®^ test [13] on several Gram-positive and Gram-negative anaerobes. The MICRONAUT-S Anaerobes^®^ MIC test has been compared to the GDM with 300 Gram-positive and Gram-negative isolates [14].

The aim of this study was to compare the two commercially available BMD kits and the GDM using the ADM as a reference after 24 and 48 h of incubation for clinically relevant bacteria of the *Clostridiales* order.

## 2. Results

### 2.1. Isolates Description

**Figure 1:**
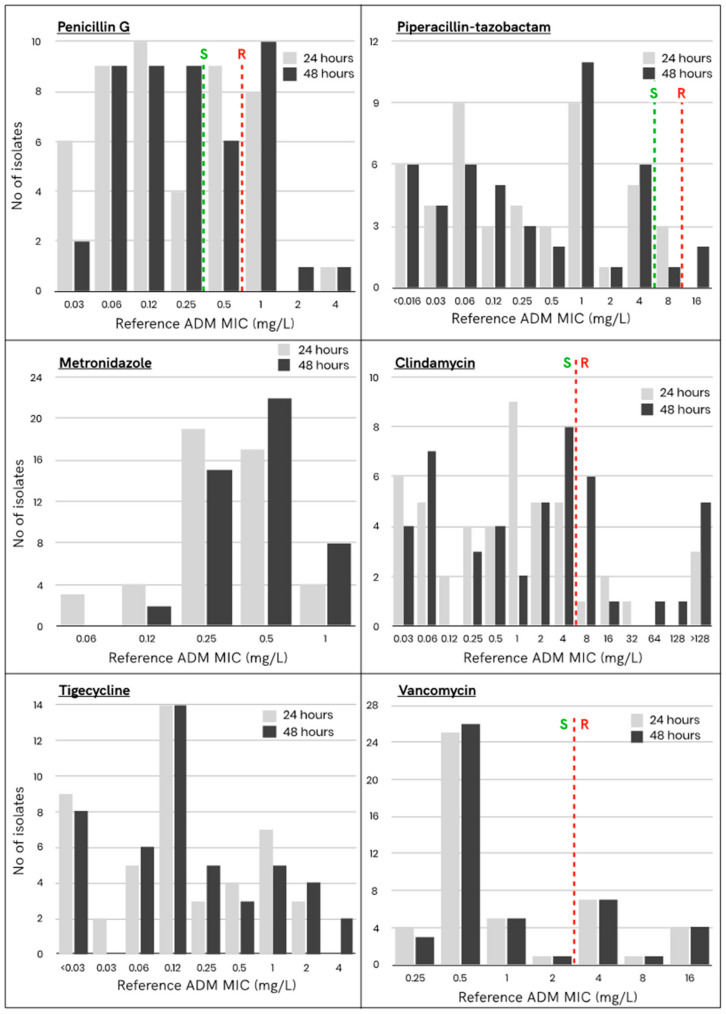
MICs distribution obtained at 24 and 48 h by the reference agar dilution method among the tested isolates and the EUCAST clinical breakpoints. The *x*-axis represents the MIC and the *y*-axis represents the number of strains with a given MIC. S: Susceptible, R: Resistant.

A total of 47 non-duplicate, clinically relevant bacterial isolates belonging to the *Clostridiales* order (genus: *Clostridium*, *Clostridioides* and *Hungatella*) were included in this study. Table 1 details the species distribution of the studied isolates as well as the clinical samples that allowed their isolation. The MICs obtained by the reference ADM after 24 h and 48 h of incubation are shown in Figure 1 and detailed in Table S1. The MICs obtained after 48 h of incubation were higher than those obtained after 24 h of incubation (Table S1). For penicillin G, one isolate had more than one dilution difference; for piperacillin/tazobactam, two isolates had more than one dilution difference; for metronidazole and tigecycline, one isolate had two dilutions of difference. This phenomenon was particularly important for clindamycin, for which 11 isolates with two or more dilutions of difference (up to nine dilutions of difference) were found. This led to a change in categorization for seven isolates using the EUCAST breakpoint.

Resistance to clindamycin was the most frequent (30% after 48 h of incubation) and was mainly observed in isolates of *C. ramosum* (*n* = 4/6), *C. innocuum* (*n* = 3/5) and *C. difficile* (*n* = 4/6). Resistance to vancomycin together with resistance to penicillin G were also frequently observed (25%) after the resistance to clindamycin. Natural resistance to vancomycin accounted for elevated MICs in the *C. ramosum* isolates (*n* = 6, MICs = 4 mg/L) and in *C. innocuum* isolates (one isolate with a MIC = 8 mg/L and four isolates with a MIC = 16 mg/L). Interestingly, one out of six *C. difficile* isolate had a vancomycin MIC of 4 mg/L which categorized it as resistant. Five out of six *C. difficile* isolates were resistant to penicillin G with MICs of 1 mg/L. Penicillin G MICs for *C. innocuum* (*n* = 3) and *C. ramosum* (*n* = 3) were above the inferior breakpoint of 0.25 mg/L. For piperacillin-tazobactam, two isolates (*C. difficile* and *C. tertium*) had a high MIC (MIC = 16 mg/L). No resistance to metronidazole was observed among these isolates.

### 2.2. Analytical Evaluation of the Two BMD Kits and the GDM

One isolate of *C. symbiosum* (2%) did not grow on any of the media used by the three techniques applied for antimicrobial susceptibility testing (GDM and the two BMD). Growth was observed for the rest of the 46 isolates after 24 h of incubation for both the MICRONAUT-S Anaerobe MIC and the MIC Test Strip. The Sensititre Anaerobe MIC plates could only be read after 48 h of incubation. After 48 h of incubation, the MICs obtained by ADM were higher compared to the MICs obtained by the two BMD methods for penicillin G and piperacillin/tazobactam, whereas, for metronidazole, the MICs were lower with ADM than with the two BMD. Similar differences were also observed with the GDM (MIC Test Strip). Clindamycin MICs were also underestimated by the Sensititre Anaerobe MIC technic. Figure S1 highlights these differences.

Very major error (VME), major error (ME), and minor error (mE) rates, and the categorical agreements (CA) and essential agreements (EA) for the MICRONAUT-S Anaerobe MIC, the Sensititre Anaerobe MIC, and for the MIC Test Strip, are shown in Table 2. For the MICRONAUT-S Anaerobe MIC, the highest values of EA were observed after 48 h of incubation. After this incubation period, EAs that were acceptable [15] for piperacillin/tazobactam, vancomycin and CA were above the FDA threshold of 90% [15] for piperacillin/tazobactam, metronidazole and vancomycin. For vancomycin, one isolate was found to be falsely susceptible (1 VME) and corresponded to a *C. ramosum* isolate which is naturally resistant to this molecule. For the Sensititre Anaerobe MIC, EA was acceptable for vancomycin, tigecycline and for piperacillin/tazobactam, but CA above the FDA threshold were observed for piperacillin/tazobactam, metronidazole and vancomycin. The three VME observed were for two *C. ramosum* and one *C. difficile* isolates. For GDM, EA values were lower than for BMD for piperacillin/tazobactam and for tigecycline and acceptable only for vancomycin. The CA were above the FDA threshold except for clindamycin and penicillin G.

## 3. Discussion

In this study, we evaluated three commercially available kits to determine the MICs of bacteria of the *Clostridiales* order (genus: *Clostridium*, *Clostridioides* and *Hungatella*) to various antibiotics. Up to now, the MICRONAUT-S Anaerobe MIC have been validated for several anaerobes species whereas the Sensititre Anaerobe MIC have been validated only for bacteria of the *Bacteroides* spp species by the manufacturer. Even though the latter represents the most commonly isolated anaerobic bacteria, bacteria of the *Clostridiales* order are also frequently isolated, and AST is recommended to be performed on them, especially for severe infections [2]. The GDM is a popular, practical and widely used tool, and the BMD is more and more often found in clinical microbiology laboratories. However, to our knowledge, this is the first study comparing the GDM and the two BDM kits to the reference ADM.

Antimicrobial resistance seems to be increasing among anaerobic bacteria, whilst antibiotic resistance patterns are becoming more and more unpredictable [5]. This phenomenon is particularly well documented for *Bacteroides* spp, for which multidrug resistant isolates have already been isolated [16,17]. Antimicrobial susceptibility of *Clostridiales* species can widely vary depending on the species. Although *C. perfringens* has important virulence factors [18], it remains susceptible to several antimicrobial agents, especially β-lactams [19,20]. In our study, no *C. perfringens* isolates were found to be resistant to penicillin G, but five were found to have elevated MICs to penicillin G which can be explained by PLP modifications as it has recently been demonstrated by Park et al. [21]. *C. ramosum*, *C. innocuum* and *C. clostridioforme* are usually more resistant to antibiotics and more particularly to β-lactams [19,22]. PLP modification seems to be the most frequent resistance mechanism, but beta-lactamases production can also be observed [22]. Resistance to clindamycin is the most frequently observed resistance among the *Clostridiales* species [19]. This resistance is particularly frequent in *C. ramosum* isolates [19] and *C. difficile* isolates [23]. Our study confirms these data and we also found *C. innocuum* clindamycin-resistant isolates. For vancomycin, elevated MICs were found for *C. ramosum* and *C. innocuum*, which is natural for these species [24,25]. Conversely, resistance to vancomycin is barely described for *C. difficile* [26]. Further specific studies are needed to determine the frequency of this resistance to a first-line treatment of *C. difficile* infections [27]. For tigecycline, the two BMD used in this study do not allow us to determine MICs lower than 1 mg/L. Consequently, taking into account the EUCAST PK-PD breakpoint of 0.25 mg/L, some resistance could not be detected. However, resistance to tigecycline is very rare in *Clostridiales* species [28].

Generally speaking, MICs were higher after 48 h of incubation, leading to change in clinical categorization. This phenomenon is particularly important for clindamycin, indeed inducible resistance can only be expressed after 48 h of incubation, which justifies the recommendation to read MICs after this time period [6,7].

Concerning analytical evaluation of the three methods. The GDM is deemed as being correlated with the reference ADM [2], but the criteria used for the evaluation are not always as stringent as those proposed by the FDA; indeed, the authors also calculated the agreement for 2 dilutions of difference [10,11]. Moreover, depending on the molecule, the GDM is not always reliable, as has already been shown by EUCAST [29,30]. For anaerobes, similar results to our study have already been found by Rennie et al. [31]: low EA (below 80% for amoxicillin/clavulanate, imipenem, metronidazole and penicillin) and CA from 82 to 92% for the same molecules were found. In a similar way to Rennie et al. [31], we found lower MICs for the tested GDM than for the reference ADM in some isolates of anaerobes. Compared to ADM, a recent report by Rentenaar et al. also pointed out low CA results with the gradient strip tests for the association amoxicillin/clavulanic acid [32]. None of the three tested methods fulfilled the FDA acceptance criteria for the six tested molecules. Clindamycin is the antimicrobial molecule for which discordances were frequently observed in the three tested techniques. Many isolates had MICs close to the clinical breakpoint of 4 mg/L, which could explain these categorization discordances. This phenomenon was also observed for penicillin G which has low clinical breakpoints. Accordingly, implementing an area of technical uncertainty could be of interest for these molecules, as it has already been proposed for other antibacterial/bacteria couples [33]. Nevertheless, piperacillin/tazobactam EAs for the two BMD kits are above the FDA’s threshold and vancomycin has a high EA value for the three tested methods. This could be of interest as these two molecules can be monitored in patients’ blood and vancomycin has a narrow therapeutic index [34]. For the three tested methods, CA fulfilled the FDA criteria for three key molecules in the treatment of anaerobic infections (metronidazole, piperacillin/tazobactam and vancomycin). In contrast, studies that have evaluated these two BMD kits to date showed more promising results [12,14], but none of them have performed the ADM method as a reference.

The relatively small number of tested isolates and the monocentric nature of our study are two limitations. Our results could be confirmed by other larger scale and multicenter studies. Moreover, our study design does not allow us to fully compare the results of our study with the literature. Indeed, in many studies, the ME and VME rates are expressed using the total number of isolates which artificially reduces these two rates.

## 4. Materials and Methods

### 4.1. Bacterial Isolates

Clinical consecutive *Clostridiales* isolates were isolated from blood cultures, deep collections and stools, (for *C. difficile* only) and were stored (−80 °C) at the bacteriology laboratory of Strasbourg University Hospital from January 2017 to July 2019 (Table 1). Identification to the species level was performed by MALDI-TOF MS with a Microflex system, according to the manufacturer’s instructions. Briefly, a colony was deposited as a thin film directly on the MALDI-TOF MS target steel plate and covered with HCCA (Alpha-cyano-4-hydroxycinnamic acid) solution. To control matrix quality, sample loading, and MALDI-TOF MS device performances, the matrix solution was deposited on each MALDI-TOF MS plate with and without bacterial control (*Pseudomonas aeruginosa* ATCC 27853). Samples were dried and analyzed by the Microflex LT MALDI-TOF MS Mass Spectrometer (Bruker Daltonics, Bremen, Germany), with detection in the linear positive-ion mode at a laser frequency of 50 Hz within a mass range of 2–20 kDa. The acceleration voltage was 20 kV, and the extraction delay time was 200 ns. Each spectrum corresponded to ions obtained from 240 laser shots performed in six regions of the same spot and was automatically acquired using the AutoXecute method with the default parameters of the flexControl v3.4 software (Bruker Daltonics, Bremen, Germany). Spectra were compared to the MBT database with 7311 MSP (Bruker Daltonics, Bremen, Germany) using the MALDI biotyper Compass Explorer v4.1.70. The reliability of species identification was estimated using the log score values (LSVs) obtained from the MALDI-Biotyper software, which ranged from 0 to 3. An LSV of at least 2 must be obtained to be considered reliable for species identification. Moreover, 0.2 minimum difference between the score of the best species match and the second species match score was required.

*B. fragilis* ATCC 25285 was used as a quality control in each experiment. MICs were compared to the manufacturer’s instructions for the BMD plates and to the CLSI official QC range [6].

### 4.2. Antimicrobial Susceptibility Testing Methods

The isolates were subcultured on Schaedler agar plates prior to AST and identification was checked again from the subculture.

#### Broth Microdilution Methods

All isolates were then tested with two BMD panels in parallel: MICRONAUT-S Anaerobes^®^ MIC test (Merlin Diagnostika GmbH, Bornheim, Germany) and Thermo Scientific Sensititre Anaerobe MIC^®^ plate (Trek Diagnostic Systems, Thermo Fisher Scientific, Cleveland, Ohio, USA) according to the manufacturer’s instructions. These two BMD panels are designed for *Bacteroides* spp AST and contained several freeze-dried antimicrobial molecules. MIC tests were performed according to the manufacturer’s instructions. The MICRONAUT-S Anaerobes^®^ MIC included 13 antibiotics. For each isolate, a 0.5 McFarland suspension was diluted in a MICRONAUT Wilkins–Chalgren in a pre-reduced broth tube (Merlin Diagnostika GmbH, Bornheim, Germany) and 100 µL of the dilution were inoculated into each well. The Sensititre Anaerobe MIC^®^ plate contained 14 antibiotics. For each bacterium, the same 0.5 McFarland suspension was diluted in a pre-reduced Brucella agar broth and 100 µL of the dilution were inoculated into each well.

#### MIC Gradient Tests

For the 6 molecules in common in both BMD kits (penicillin G, piperacillin/tazobactam, metronidazole, clindamycin, tigecycline and vancomycin), gradient diffusion method was also performed using the MIC Test Strip (Liofilchem^®^, Waverley, MA, USA). These latter were tested on Brucella agar plate with K1 vitamin, hemin and 5% sheep blood (BD, Franklin Lakes, USA) using a 1 MacFarland suspension according to the manufacturer’s instructions. Plates were incubated under anaerobic conditions in an anaerobic chamber. According to the manufacturer’s instructions, MICs were read at the intersection of the inhibition ellipse with the strip.

The two BMD and the gradient tests were incubated under anaerobic conditions in an anaerobic chamber (Whitley DG1000 anaerobic workstation, Don Whitley Scientific, Bingley, England). They were read after 24 and 48 h of incubation at 35 +/− 2 °C.

#### Agar Dilution Method

For the same 6 molecules, reference MICs were determined using agar dilution method (ADM) according to the CLSI recommendations [6]. Briefly, in-house Brucella agar plates (with K1 vitamin, hemin and 5% sheep blood) to which antimicrobial molecules were added were prepared (BBL Brucella agar – BD, Franklin Lakes, NJ, USA). For each antibiotic, antibiotic dilutions were tested (from 0.016 to 128 mg/L for penicillin G, piperacillin/tazobactam and clindamycin-from 0.03 to 128 mg/L for tigecycline, metronidazole and vancomycin). Then, 1 µL of a 0.5 McFarland bacterial suspension was inoculated with Steers’ replicator (Denley, England). Plates were incubated under anaerobic conditions using jars and an anoxomat system (I&L Biosystems, Königswinter, Germany).

### 4.3. MIC Interpretation

For all the methods performed in this study, MICs were interpreted with the EUCAST breakpoints [35] for Gram-positive anaerobes, expect for tigecycline, for which there is no clinical breakpoint, hence essential agreement was only evaluated.

Discordant results between a tested method and the reference ADM were repeated once.

### 4.4. Evaluation of Analytical Performances

Performances of the Sensititre Anaerobes MIC^®^ plate, the MICRONAUT-S Anaerobe MIC^®^ test, and the gradient diffusion method were compared to the ADM reference method. The growth failure rate was reported, and for each molecule, the categorical and essential agreements were evaluated according to the Food and Drug Administration (FDA) recommendations [15]. The essential agreement (EA) is defined by the FDA as ‘agreement within plus or minus, one two-fold dilution of the new device under evaluation with the reference method MIC’. The EA rate was expressed by the ratio of isolates in EA on the total number of bacterial isolates tested. The categorical agreement (CA) is defined as ‘agreement of interpretive results (Susceptible—S, Intermediate—I, and Resistant—R) between a new device under evaluation and a standard reference method’. The CA rate was expressed by the ratio of isolates with the same clinical category (SIR) as the reference method on the total number of bacterial isolates tested. Discrepancies were categorized in minor error (mE), major error (ME) and very major error (VME). The mE rate was calculated by the ratio of isolates categorized S or R by the reference method and interpreted as I with the evaluated device on the total number of bacterial isolates tested. The ME rate was calculated by the ratio of isolates categorized S by the reference method and interpreted as R with the evaluated device on the number of susceptible bacterial isolates as determined by the reference method. Finally, the VME rate was calculated by the ratio of isolates categorized as R by the reference method but interpreted as S with the evaluated device based on the number of resistant bacterial isolates as determined by the reference method. All rates were expressed in percentages.

According to the FDA [15], the growth failure rate must be <10%, CA and EA must be ≥ 90% and the ME rate ≤3% and VME must be ≤1.5%.

## 5. Conclusions

Broth microdilution is a handy and promising method for MIC determination, this method is recommended for *Bacteroides* spp. by the CLSI [6]. Based on the results of this study, analytical performances of the two BMD techniques and the GDM vary according to the studied molecules for the *Clostridiales* species. The EA varied according to the molecule and the considered kit. For the three tested methods, the CA fulfilled the FDA criteria for three key molecules in the treatment of anaerobic infections (metronidazole, piperacillin/tazobactam and vancomycin). Nevertheless, the clindamycin CA rate was below 90%, regardless of the kit evaluated.

## Figures and Tables

**Table 1:** Species distribution of the isolates used for this study and their origins.

Species	Number of Isolates	Blood Culture	Stool	Deep Abscess	Peritoneal Fluid
*C. perfringens*	17	15	-	2	-
*C. difficile*	6	-	6	-	-
*C. ramosum*	6	6	-	-	-
*C. septicum*	6	6	-	-	-
*C. innocuum*	5	4	-	-	1
*C. citroniae*	2	2	-	-	-
*C. paraputrificum*	1	1	-	-	-
*C. barati*	1	1	-	-	-
*C. tertium*	1	1	-	-	-
*C. symbiosum*	1	1	-	-	-
*H. hathewayi*	1	1	-	-	-
Total	47				

**Table 2:** Percentage and number of essential or categorical errors for the three methods evaluated in this study according to the reading time for the 46 studied isolates using ADM as reference method. EA: essential agreement; CA: categorical agreement; mE: minor error; ME: major error; VME: very major error.

Molecules	No. of Isolates in Each Category			EA % (n)	CA % (n)	mE % (n)	ME % (n)	VME % (n)	EA % (n)	CA % (n)	mE % (n)	ME % (n)	VME % (n)	EA % (n)	CA % (n)	mE % (n)	ME % (n)	VME % (n)
	S	I	R	MICRONAUT-S Anaerobe MIC (24 h)					Sensititre Anaerobe MIC (24 h)					MIC Test Strip (24 h)				
Penicillin G	29	9	8	76.1 (35)	69.6 (32)	26.1% (12)	0% (0)	25% (2)	-	-	-	-	-	82.6% (38)	82.6% (38)	15.2% (7)	0% (0)	12.5% (1)
Piperacillin/tazobactam	46	0	0	87% (40)	100% (46)	-	0% (0)	-	-	-	-	-	-	71.7% (33)	100% (46)	-	0% (0)	-
Metronidazole	46	-	0	69.6% (32)	97.8% (45)	-	2.2% (1)	-	-	-	-	-	-	60.8% (28)	95.65% (44)	-	4.35% (2)	-
Clindamycin	39	-	7	63% (29)	84.8% (39)	-	10.3% (4)	42.9% (3)	-	-	-	-	-	65.2% (30)	87.9% (40)	-	10.2% (4)	28.6% (2)
Tigecycline	-	-	-	80.8% (38)	-	-	-	-	-	-	-	-	-	71.7% (33)	-	-	-	-
Vancomycin	34	-	12	95.7% (44)	93.4% (43)	-	0%(0)	25% (3)	-	-	-	-	-	97.9% (45)	97.9% (45)	-	0%(0)	8.33% (1)
	**S**	**I**	**R**	**MICRONAUT-S Anaerobe MIC (48 h)**					**Sensititre Anaerobe MIC (48 h)**					**MIC Test Strip (48 h)**				
Penicillin G	29	6	11	71.7% (33)	76.1% (35)	19.5% (9)	0% (0)	18.2% (2)	87% (40)	82.6% (38)	15.2% (7)	0% (0)	9.1% (1)	76.1% (35)	84.8% (39)	13.0% (6)	0% (0)	9.1% (1)
Piperacillin/tazobactam	44	2	0	91.3% (42)	95.6% (44)	4.35% (2)	0% (0)	-	91.3% (42)	97.8% (45)	2.13% (1)	0% (0)	-	69.1% (32)	95.6% (44)	4.35% (2)	0% (0)	-
Metronidazole	46	-	0	69.6% (32)	97.8% (45)	-	2.2% (1)	-	43.5% (20)	95.6% (44)	-	4.35% (2)	-	71.7% (33)	95.6% (44)	-	4.35% (2)	-
Clindamycin	32	-	14	84.7% (39)	82.6% (38)	-	12.5% (4)	28.6% (4)	87% (40)	89.1% (41)	-	6.25% (2)	21.4% (3)	76.1% (35)	80.4% (37)	-	6.25% (2)	50% (7)
Tigecycline	-	-	-	82.6% (38)	-	-	-	-	93.4% (43)	-	-	-	-	65.2% (30)	-	-	-	-
Vancomycin	34	-	12	97.8% (45)	97.8% (45)	-	0%(0)	8.33% (1)	97.9% (45)	93.4% (43)	-	0% (0)	25% (3)	97.9% (45)	97.9% (45)	-	0% (0)	8.33% (1)

## Data Availability

All data generated or analyzed during this study have been included in this article.

## Supplementary Materials

The following are available online at https://www.mdpi.com/article/10.3390/antibiotics10080975/s1, Figure S1: correlation tables obtained for the three evaluated methods after 48 h of incubation. Table S1: MALDI-TOF MS results and MICs and clinical categorization obtained after 24 and 48 h of incubation by the reference ADM.

## References

[1] Nagy E., Boyanova L., Justesen U.S. (2018). How to isolate, identify and determine antimicrobial susceptibility of anaerobic bacteria in routine laboratories. Clin. Microbiol. Infect..

[2] Brook I., Wexler H.M., Goldstein E.J.C. (2013). Antianaerobic Antimicrobials: Spectrum and Susceptibility Testing. Clin. Microbiol. Rev..

[3] Brook I. (2007). Anaerobic Infections: Diagnosis and Management.

[4] Salonen J.H., Eerola E., Meurman O. (1998). Clinical Significance and Outcome of Anaerobic Bacteremia. Clin. Infect. Dis..

[5] Boyanova L., Kolarov R., Mitov I. (2015). Recent evolution of antibiotic resistance in the anaerobes as compared to previous decades. Anaerobe.

[6] (2021). CLSI M100-ED31:2021 Performance Standards for Antimicrobial Susceptibility Testing.

[7] (2021). CASFM/EUCAST: Recommandations 2021.

[8] Torres E., Delgado-Valverde M., Valiente A., Pascual Á., Rodríguez-Baño J. (2015). Impact of borderline minimum inhibitory concentration on the outcome of invasive infections caused by Enterobacteriaceae treated with β-lactams: A systematic review and meta-analysis. Eur. J. Clin. Microbiol. Infect. Dis..

[9] Poulet P.P., Duffaut D., Lodter J.P. (1999). Evaluation of the Etest for determining the in-vitro susceptibilities of Prevotella intermedia isolates to metronidazole. J. Antimicrob. Chemother..

[10] Croco J.L., Erwin M.E., Jennings J.M., Putnam L.R., Jones R.N. (1994). Evaluation of the etest for antimicrobial spectrum and potency determinations of anaerobes associated with bacterial vaginosis and peritonitis. Diagn. Microbiol. Infect. Dis..

[11] Citron D.M., Ostovari M.I., Karlsson A., Goldstein E.J. (1991). Evaluation of the E test for susceptibility testing of anaerobic bacteria. J. Clin. Microbiol..

[12] Hughes C., Ashhurst-Smith C., Ferguson J. (2018). Gram negative anaerobe susceptibility testing in clinical isolates using Sensititre and Etest methods. Pathology.

[13] Cherkaoui A., Fischer A., Azam N., Riat A., Schrenzel J. (2018). A comparison of Sensititre ™ Anaerobe MIC plate with ATB ANA^®^ test for the routine susceptibility testing of common anaerobe pathogens. Eur. J. Clin. Microbiol. Infect. Dis..

[14] Cordovana M., Ambretti S. (2020). Antibiotic susceptibility testing of anaerobic bacteria by broth microdilution method using the MICRONAUT-S Anaerobes MIC plates. Anaerobe.

[15] Antimicrobial Susceptibility Test (AST) Systems—Class ii Special Controls Guidance for Industry and FDA, 2018. FDA. https://www.fda.gov/medical-devices/guidance-documents-medical-devices-and-radiation-emitting-products/antimicrobial-susceptibility-test-ast-systems-class-ii-special-controls-guidance-industry-and-fda.

[16] Nagy E., Urbán E., Nord C.E. (2011). Antimicrobial susceptibility of Bacteroides fragilis group isolates in Europe: 20 years of experience. Clin. Microbiol. Infect..

[17] Kaeuffer C., Ruge T., Diancourt L., Romain B., Ruch Y., Jaulhac B., Boyer P. (2021). First Case of Bacteraemia Due to Carbapenem-Resistant. Antibiotics.

[18] Yao P., Annamaraju P. (2021). Clostridium perfringens. StatPearls.

[19] Forbes J.D., Kus J.V., Patel S.N. (2021). Antimicrobial susceptibility profiles of invasive isolates of anaerobic bacteria from a large Canadian reference laboratory: 2012–2019. Anaerobe.

[20] EUCAST Antimicrobial Wild Type Distributions of Microorganisms. https://mic.eucast.org/.

[21] Park M., Rafii F. (2017). Exposure to β-lactams results in the alteration of penicillin-binding proteins in Clostridium perfringens. Anaerobe.

[22] Alexander C.J., Citron D.M., Brazier J.S., Goldstein E.J. (1995). Identification and antimicrobial resistance patterns of clinical isolates of Clostridium clostridioforme, Clostridium innocuum, and Clostridium ramosum compared with those of clinical isolates of Clostridium perfringens. J. Clin. Microbiol..

[23] Jon J.V., Mark H.W., Jane F. (2021). Antimicrobial resistance progression in the United Kingdom: A temporal comparison of Clostridioides difficile antimicrobial susceptibilities. Anaerobe.

[24] Tally F.P., Armfield A.Y., Dowell V.R., Kwok Y.-Y., Sutter V.L., Finegold S.M. (1974). Susceptibility of Clostridium ramosum to Antimicrobial Agents. Antimicrob. Agents Chemother..

[25] David V., Bozdogan B., Mainardi J.-L., Legrand R., Gutmann L., Leclercq R. (2004). Mechanism of Intrinsic Resistance to Vancomycin in Clostridium innocuum NCIB 10674. J. Bacteriol..

[26] Peláez T., Alcalá L., Alonso R., Rodríguez-Créixems M., García-Lechuz J.M., Bouza E. (2002). Reassessment of Clostridium difficile Susceptibility to Metronidazole and Vancomycin. Antimicrob. Agents Chemother..

[27] Ooijevaar R., van Beurden Y., Terveer E., Goorhuis A., Bauer M., Keller J., Mulder C., Kuijper E. (2018). Update of treatment algorithms for Clostridium difficile infection. Clin. Microbiol. Infect..

[28] Hawser S.P. (2010). Activity of tigecycline against multidrug-resistant clinical isolates of Clostridium spp. from Europe. Int. J. Antimicrob. Agents.

[29] Matuschek E., Åhman J., Webster C., Kahlmeter G. (2018). Antimicrobial susceptibility testing of colistin—Evaluation of seven commercial MIC products against standard broth microdilution for Escherichia coli, Klebsiella pneumoniae, Pseudomonas aeruginosa, and *Acinetobacter* spp. Clin. Microbiol. Infect..

[30] EUCAST (2019). EUCAST Warning against the Use of Gradient Tests for Benzylpenicillin MIC in Streptococcus Pneumoniae.

[31] Rennie R.P., Turnbull L., Brosnikoff C., Cloke J. (2012). First Comprehensive Evaluation of the M.I.C. Evaluator Device Compared to Etest and CLSI Reference Dilution Methods for Antimicrobial Susceptibility Testing of Clinical Strains of Anaerobes and Other Fastidious Bacterial Species. J. Clin. Microbiol..

[32] Rentenaar R.J., Bovo-Heijmans B., Diggle J., Fluit A.C., Wootton M. (2021). False amoxicillin/clavulanic acid susceptibility in Bacteroides fragilis using gradient strip tests. Anaerobe.

[33] Soares A., Pestel-Caron M., De Rohello F.L., Bourgoin G., Boyer S., Caron F. (2020). Area of technical uncertainty for susceptibility testing of amoxicillin/clavulanate against Escherichia coli: Analysis of automated system, Etest and disk diffusion methods compared to the broth microdilution reference. Clin. Microbiol. Infect..

[34] Lodise T.P., Patel N., Lomaestro B.M., Rodvold K.A., Drusano G.L. (2009). Relationship between Initial Vancomycin Concentration-Time Profile and Nephrotoxicity among Hospitalized Patients. Clin. Infect. Dis..

[35] EUCAST (2020). The European Committee on Antimicrobial Susceptibility Testing. Breakpoint Tables for Interpretation of MICs and Zone Diameters.

