# Supplementary material for: Broth Microdilution and Gradient Diffusion Strips vs. Reference Agar Dilution Method: First Evaluation for Clostridiales Species Antimicrobial Susceptibility Testing
Source: Antibiotics (Basel). 2021 Aug 12;10(8):975. doi: 10.3390/antibiotics10080975 (PMC8388896; doi:10.3390/antibiotics10080975)
Supplement: Supplementary file 1 [file antibiotics-10-00975-s001.zip › Supplementary files/Add file Figure S1.pptx]

## Slide 1
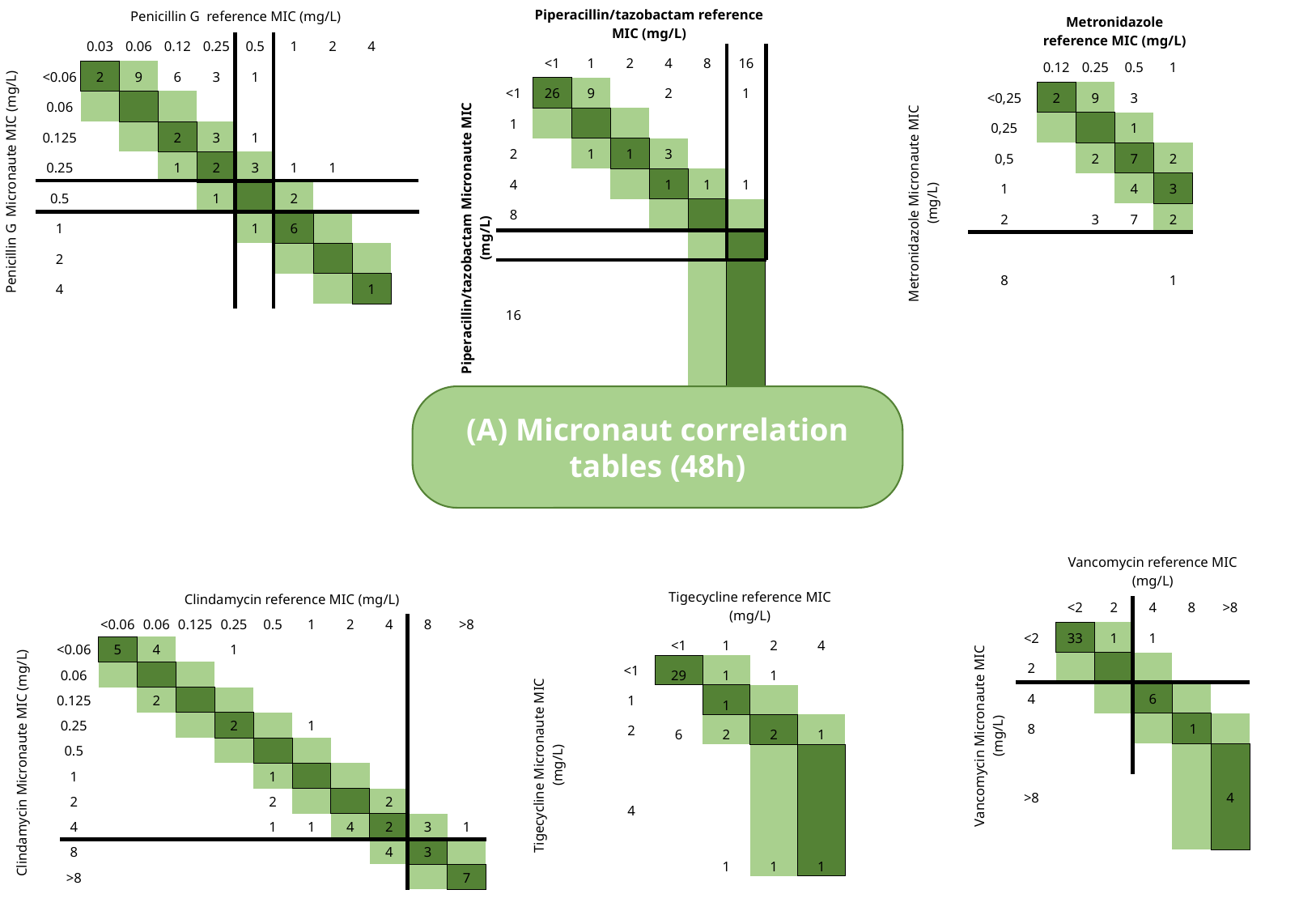

| | | Penicillin G reference MIC (mg/L) | | | | | | | |
| --- | --- | --- | --- | --- | --- | --- | --- | --- | --- |
| | | 0.03 | 0.06 | 0.12 | 0.25 | 0.5 | 1 | 2 | 4 |
| Penicillin G Micronaute MIC (mg/L) | <0.06 | 2 | 9 | 6 | 3 | 1 | | | |
| | 0.06 | | | | | | | | |
| | 0.125 | | | 2 | 3 | 1 | | | |
| | 0.25 | | | 1 | 2 | 3 | 1 | 1 | |
| | 0.5 | | | | 1 | | 2 | | |
| | 1 | | | | | 1 | 6 | | |
| | 2 | | | | | | | | |
| | 4 | | | | | | | | 1 |
| | | Piperacillin/tazobactam reference MIC (mg/L) | | | | | |
| --- | --- | --- | --- | --- | --- | --- | --- |
| | | <1 | 1 | 2 | 4 | 8 | 16 |
| Piperacillin/tazobactam Micronaute MIC (mg/L) | <1 | 26 | 9 | | 2 | | 1 |
| | 1 | | | | | | |
| | 2 | | 1 | 1 | 3 | | |
| | 4 | | | | 1 | 1 | 1 |
| | 8 | | | | | | |
| | 16 | | | | | | |
| | | Metronidazole reference MIC (mg/L) | | | |
| --- | --- | --- | --- | --- | --- |
| | | 0.12 | 0.25 | 0.5 | 1 |
| Metronidazole Micronaute MIC (mg/L) | <0,25 | 2 | 9 | 3 | |
| | 0,25 | | | 1 | |
| | 0,5 | | 2 | 7 | 2 |
| | 1 | | | 4 | 3 |
| | 2 | | 3 | 7 | 2 |
| | 8 | | | | 1 |
(A) Micronaut correlation tables (48h)
| | | Vancomycin reference MIC (mg/L) | | | | |
| --- | --- | --- | --- | --- | --- | --- |
| | | <2 | 2 | 4 | 8 | >8 |
| Vancomycin Micronaute MIC (mg/L) | <2 | 33 | 1 | 1 | | |
| | 2 | | | | | |
| | 4 | | | 6 | | |
| | 8 | | | | 1 | |
| | >8 | | | | | 4 |
| | | Clindamycin reference MIC (mg/L) | | | | | | | | | |
| --- | --- | --- | --- | --- | --- | --- | --- | --- | --- | --- | --- |
| | | <0.06 | 0.06 | 0.125 | 0.25 | 0.5 | 1 | 2 | 4 | 8 | >8 |
| Clindamycin Micronaute MIC (mg/L) | <0.06 | 5 | 4 | | 1 | | | | | | |
| | 0.06 | | | | | | | | | | |
| | 0.125 | | 2 | | | | | | | | |
| | 0.25 | | | | 2 | | 1 | | | | |
| | 0.5 | | | | | | | | | | |
| | 1 | | | | | 1 | | | | | |
| | 2 | | | | | 2 | | | 2 | | |
| | 4 | | | | | 1 | 1 | 4 | 2 | 3 | 1 |
| | 8 | | | | | | | | 4 | 3 | |
| | >8 | | | | | | | | | | 7 |
| | | Tigecycline reference MIC (mg/L) | | | |
| --- | --- | --- | --- | --- | --- |
| | | <1 | 1 | 2 | 4 |
| Tigecycline Micronaute MIC (mg/L) | <1 | 29 | 1 | 1 | |
| | 1 | | 1 | | |
| | 2 | 6 | 2 | 2 | 1 |
| | 4 | | 1 | 1 | 1 |

## Slide 2
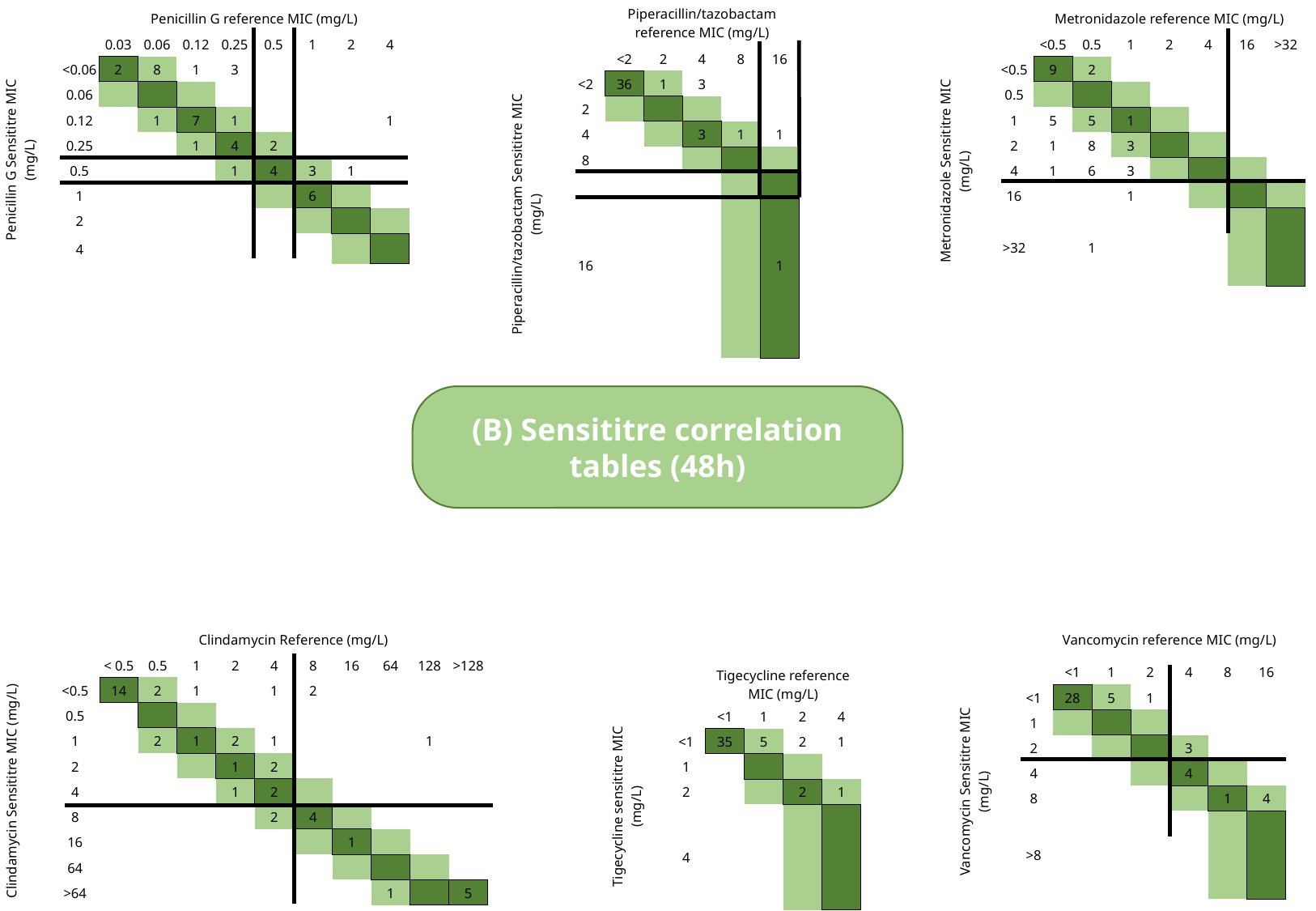

| | | Piperacillin/tazobactam reference MIC (mg/L) | | | | |
| --- | --- | --- | --- | --- | --- | --- |
| | | <2 | 2 | 4 | 8 | 16 |
| Piperacillin/tazobactam Sensititre MIC (mg/L) | <2 | 36 | 1 | 3 | | |
| | 2 | | | | | |
| | 4 | | | 3 | 1 | 1 |
| | 8 | | | | | |
| | 16 | | | | | 1 |
| | | Penicillin G reference MIC (mg/L) | | | | | | | |
| --- | --- | --- | --- | --- | --- | --- | --- | --- | --- |
| | | 0.03 | 0.06 | 0.12 | 0.25 | 0.5 | 1 | 2 | 4 |
| Penicillin G Sensititre MIC (mg/L) | <0.06 | 2 | 8 | 1 | 3 | | | | |
| | 0.06 | | | | | | | | |
| | 0.12 | | 1 | 7 | 1 | | | | 1 |
| | 0.25 | | | 1 | 4 | 2 | | | |
| | 0.5 | | | | 1 | 4 | 3 | 1 | |
| | 1 | | | | | | 6 | | |
| | 2 | | | | | | | | |
| | 4 | | | | | | | | |
| | | Metronidazole reference MIC (mg/L) | | | | | | |
| --- | --- | --- | --- | --- | --- | --- | --- | --- |
| | | <0.5 | 0.5 | 1 | 2 | 4 | 16 | >32 |
| Metronidazole Sensititre MIC (mg/L) | <0.5 | 9 | 2 | | | | | |
| | 0.5 | | | | | | | |
| | 1 | 5 | 5 | 1 | | | | |
| | 2 | 1 | 8 | 3 | | | | |
| | 4 | 1 | 6 | 3 | | | | |
| | 16 | | | 1 | | | | |
| | >32 | | 1 | | | | | |
(B) Sensititre correlation tables (48h)
| | | Vancomycin reference MIC (mg/L) | | | | | |
| --- | --- | --- | --- | --- | --- | --- | --- |
| | | <1 | 1 | 2 | 4 | 8 | 16 |
| Vancomycin Sensititre MIC (mg/L) | <1 | 28 | 5 | 1 | | | |
| | 1 | | | | | | |
| | 2 | | | | 3 | | |
| | 4 | | | | 4 | | |
| | 8 | | | | | 1 | 4 |
| | >8 | | | | | | |
| | | Clindamycin Reference (mg/L) | | | | | | | | | |
| --- | --- | --- | --- | --- | --- | --- | --- | --- | --- | --- | --- |
| | | < 0.5 | 0.5 | 1 | 2 | 4 | 8 | 16 | 64 | 128 | >128 |
| Clindamycin Sensititre MIC (mg/L) | <0.5 | 14 | 2 | 1 | | 1 | 2 | | | | |
| | 0.5 | | | | | | | | | | |
| | 1 | | 2 | 1 | 2 | 1 | | | | 1 | |
| | 2 | | | | 1 | 2 | | | | | |
| | 4 | | | | 1 | 2 | | | | | |
| | 8 | | | | | 2 | 4 | | | | |
| | 16 | | | | | | | 1 | | | |
| | 64 | | | | | | | | | | |
| | >64 | | | | | | | | 1 | | 5 |
| | | Tigecycline reference MIC (mg/L) | | | |
| --- | --- | --- | --- | --- | --- |
| Tigecycline sensititre MIC (mg/L) | | <1 | 1 | 2 | 4 |
| | <1 | 35 | 5 | 2 | 1 |
| | 1 | | | | |
| | 2 | | | 2 | 1 |
| | 4 | | | | |

## Slide 3
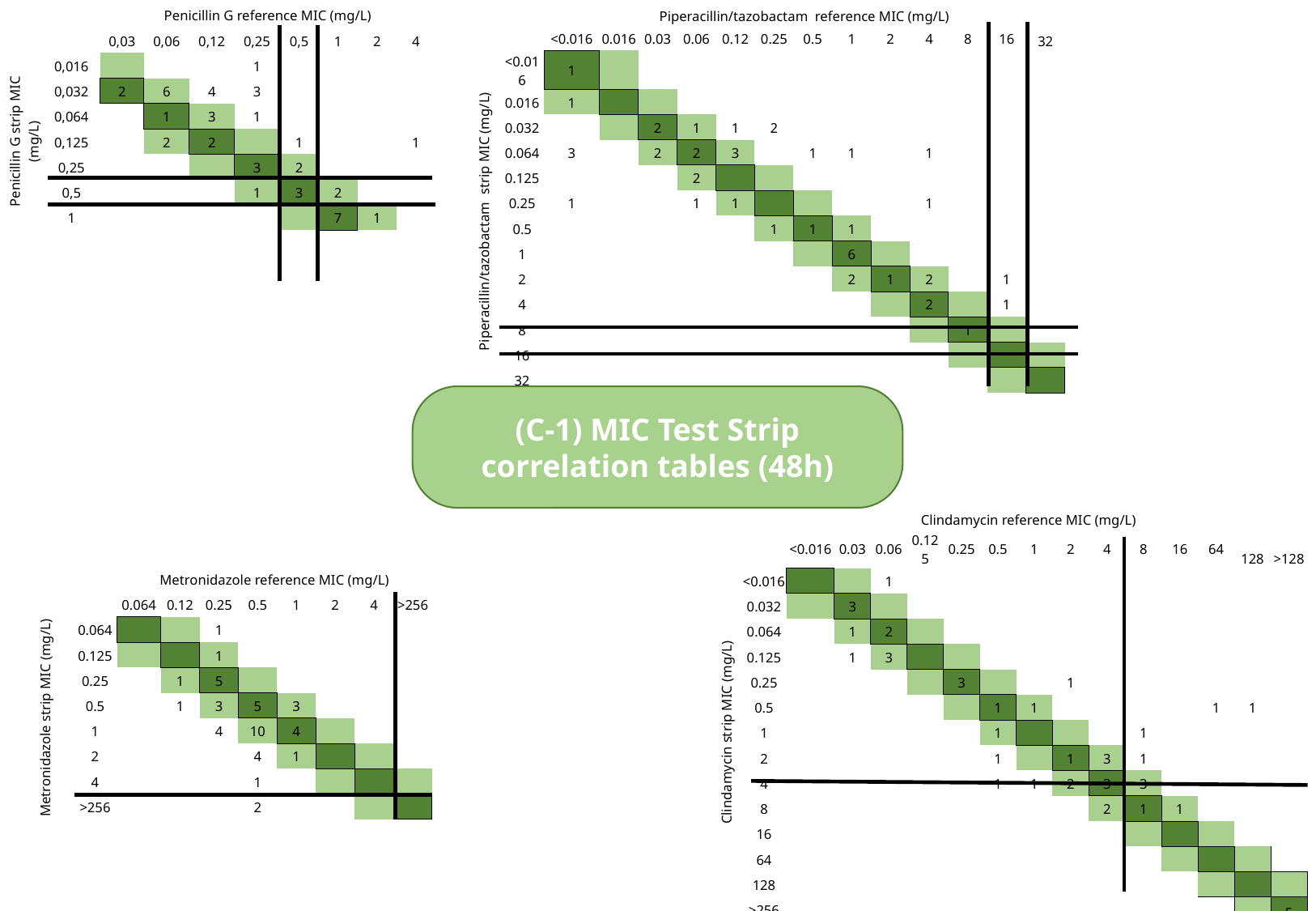

| | | Piperacillin/tazobactam reference MIC (mg/L) | | | | | | | | | | | | |
| --- | --- | --- | --- | --- | --- | --- | --- | --- | --- | --- | --- | --- | --- | --- |
| | | <0.016 | 0.016 | 0.03 | 0.06 | 0.12 | 0.25 | 0.5 | 1 | 2 | 4 | 8 | 16 | 32 |
| Piperacillin/tazobactam strip MIC (mg/L) | <0.016 | 1 | | | | | | | | | | | | |
| | 0.016 | 1 | | | | | | | | | | | | |
| | 0.032 | | | 2 | 1 | 1 | 2 | | | | | | | |
| | 0.064 | 3 | | 2 | 2 | 3 | | 1 | 1 | | 1 | | | |
| | 0.125 | | | | 2 | | | | | | | | | |
| | 0.25 | 1 | | | 1 | 1 | | | | | 1 | | | |
| | 0.5 | | | | | | 1 | 1 | 1 | | | | | |
| | 1 | | | | | | | | 6 | | | | | |
| | 2 | | | | | | | | 2 | 1 | 2 | | 1 | |
| | 4 | | | | | | | | | | 2 | | 1 | |
| | 8 | | | | | | | | | | | 1 | | |
| | 16 | | | | | | | | | | | | | |
| | 32 | | | | | | | | | | | | | |
| | | Penicillin G reference MIC (mg/L) | | | | | | | |
| --- | --- | --- | --- | --- | --- | --- | --- | --- | --- |
| | | 0,03 | 0,06 | 0,12 | 0,25 | 0,5 | 1 | 2 | 4 |
| Penicillin G strip MIC (mg/L) | 0,016 | | | | 1 | | | | |
| | 0,032 | 2 | 6 | 4 | 3 | | | | |
| | 0,064 | | 1 | 3 | 1 | | | | |
| | 0,125 | | 2 | 2 | | 1 | | | 1 |
| | 0,25 | | | | 3 | 2 | | | |
| | 0,5 | | | | 1 | 3 | 2 | | |
| | 1 | | | | | | 7 | 1 | |
(C-1) MIC Test Strip correlation tables (48h)
| | | Clindamycin reference MIC (mg/L) | | | | | | | | | | | | | |
| --- | --- | --- | --- | --- | --- | --- | --- | --- | --- | --- | --- | --- | --- | --- | --- |
| | | <0.016 | 0.03 | 0.06 | 0.125 | 0.25 | 0.5 | 1 | 2 | 4 | 8 | 16 | 64 | 128 | >128 |
| Clindamycin strip MIC (mg/L) | <0.016 | | | 1 | | | | | | | | | | | |
| | 0.032 | | 3 | | | | | | | | | | | | |
| | 0.064 | | 1 | 2 | | | | | | | | | | | |
| | 0.125 | | 1 | 3 | | | | | | | | | | | |
| | 0.25 | | | | | 3 | | | 1 | | | | | | |
| | 0.5 | | | | | | 1 | 1 | | | | | 1 | 1 | |
| | 1 | | | | | | 1 | | | | 1 | | | | |
| | 2 | | | | | | 1 | | 1 | 3 | 1 | | | | |
| | 4 | | | | | | 1 | 1 | 2 | 3 | 3 | | | | |
| | 8 | | | | | | | | | 2 | 1 | 1 | | | |
| | 16 | | | | | | | | | | | | | | |
| | 64 | | | | | | | | | | | | | | |
| | 128 | | | | | | | | | | | | | | |
| | >256 | | | | | | | | | | | | | | 5 |
| | | Metronidazole reference MIC (mg/L) | | | | | | | |
| --- | --- | --- | --- | --- | --- | --- | --- | --- | --- |
| | | 0.064 | 0.12 | 0.25 | 0.5 | 1 | 2 | 4 | >256 |
| Metronidazole strip MIC (mg/L) | 0.064 | | | 1 | | | | | |
| | 0.125 | | | 1 | | | | | |
| | 0.25 | | 1 | 5 | | | | | |
| | 0.5 | | 1 | 3 | 5 | 3 | | | |
| | 1 | | | 4 | 10 | 4 | | | |
| | 2 | | | | 4 | 1 | | | |
| | 4 | | | | 1 | | | | |
| | >256 | | | | 2 | | | | |

## Slide 4
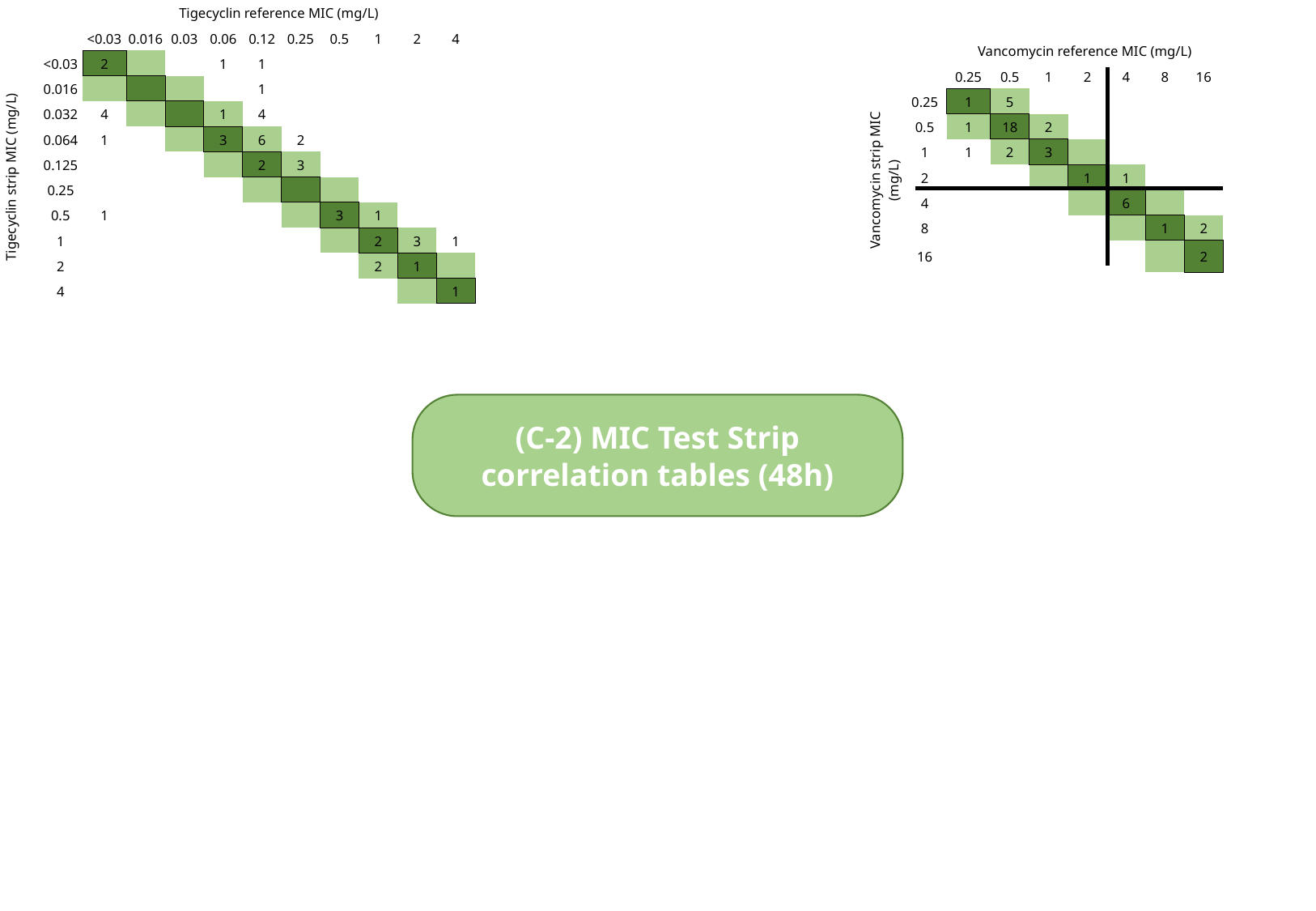

| | | Tigecyclin reference MIC (mg/L) | | | | | | | | | |
| --- | --- | --- | --- | --- | --- | --- | --- | --- | --- | --- | --- |
| | | <0.03 | 0.016 | 0.03 | 0.06 | 0.12 | 0.25 | 0.5 | 1 | 2 | 4 |
| Tigecyclin strip MIC (mg/L) | <0.03 | 2 | | | 1 | 1 | | | | | |
| | 0.016 | | | | | 1 | | | | | |
| | 0.032 | 4 | | | 1 | 4 | | | | | |
| | 0.064 | 1 | | | 3 | 6 | 2 | | | | |
| | 0.125 | | | | | 2 | 3 | | | | |
| | 0.25 | | | | | | | | | | |
| | 0.5 | 1 | | | | | | 3 | 1 | | |
| | 1 | | | | | | | | 2 | 3 | 1 |
| | 2 | | | | | | | | 2 | 1 | |
| | 4 | | | | | | | | | | 1 |
| | | Vancomycin reference MIC (mg/L) | | | | | | |
| --- | --- | --- | --- | --- | --- | --- | --- | --- |
| | | 0.25 | 0.5 | 1 | 2 | 4 | 8 | 16 |
| Vancomycin strip MIC (mg/L) | 0.25 | 1 | 5 | | | | | |
| | 0.5 | 1 | 18 | 2 | | | | |
| | 1 | 1 | 2 | 3 | | | | |
| | 2 | | | | 1 | 1 | | |
| | 4 | | | | | 6 | | |
| | 8 | | | | | | 1 | 2 |
| | 16 | | | | | | | 2 |
(C-2) MIC Test Strip correlation tables (48h)
